# Effectiveness of eHealth weight management interventions in overweight and obese adults from low socioeconomic groups: a systematic review

**DOI:** 10.1186/s13643-023-02207-3

**Published:** 2023-03-30

**Authors:** Richard Myers-Ingram, Jade Sampford, Rhian Milton-Cole, Gareth David Jones

**Affiliations:** 1 Department of Physiotherapy, Guy’s & St Thomas’ Hospital NHS Foundation Trust, London, UK; 2 Department of Population and Health Sciences, King’s College London, London, UK; 3 Faculty of Life Sciences & Medicine, Centre for Human & Applied Physiological Sciences (CHAPS), King’s College London, London, UK

**Keywords:** Obesity, eHealth, Weight management, Weight loss, Low socioeconomic status, Socioeconomic

## Abstract

**Background:**

Low socioeconomic status (SES) is associated with increased rates of overweight and obesity. Proponents of electronic health (eHealth) hypothesise that its inclusion in weight management interventions can improve efficacy by mitigating typical barriers associated with low SES.

**Objectives:**

To establish the scope of eHealth weight management interventions for people with overweight and obesity from a low SES. Secondary objectives were to determine the efficacy of eHealth interventions in facilitating weight loss, physical activity and fitness improvements.

**Methods:**

Four databases and grey literature were systematically searched to identify eligible studies published in English from inception to May 2021. Studies examining an eHealth intervention with low SES participants were included. Outcomes included temporal change in weight and BMI, anthropometry, physiological measures and physical activity levels. The number and heterogeneity of studies precluded any meta-analyses; thus, a narrative review was undertaken.

**Results:**

Four experimental studies with low risk of bias were reviewed. There was variance in how SES was defined. Study aims and eHealth media also varied and included reducing/maintaining weight or increasing physical activity using interactive websites or voice responses, periodic communication and discourse via telephone, social media, text messaging or eNewsletters. Irrespectively, all studies reported short-term weight loss. eHealth interventions also increased short-term physical activity levels where it was assessed, but did not change anthropometry or physiological measures. None reported any effect on physical fitness.

**Conclusions:**

This review revealed short-term effects of eHealth interventions on weight loss and increased physical activity levels for low SES participants. Evidence was limited to a small number of studies, with small to moderate sample sizes. Inter-study comparison is challenging because of considerable variability. Future work should prioritise how to utilise eHealth in the longer term either as a supportive public health measure or by determining its long-term efficacy in engendering volitional health behaviour changes.

**Systematic review registration:**

PROSPERO CRD42021243973

**Supplementary Information:**

The online version contains supplementary material available at 10.1186/s13643-023-02207-3.

## Introduction

Overweight and obesity, defined as abnormal or excessive fat accumulation that may impair health, are typically measured using body mass index (BMI) (the ratio of mass (kg) to squared height (m^2^)) [1]. Overweight is classified as a *BMI* ≥ 25 kg/m^2^ and obese ≥ 30 kg/m^2^ [2]. Overweight and obesity global prevalence are high [3–5], with an estimated 60% of females and 67% of males overweight or obese in England [6]. Overweight and obesity are a significant risk factor for noncommunicable diseases including type 2 diabetes, cardiovascular disease, specific cancers, liver disease and some respiratory disease [7] as well as depression [8]. Addressing overweight and obesity is therefore essential for the individual themselves, clinicians and policy makers [9, 10]. The World Health Organization (WHO) has prioritised the prevention and reduction of obesity as a key public health agenda, recommending nations make substantial improvements to tackle the current obesity trends [11].

Socioeconomic status (SES) is a complex concept involving several domains, including an individual’s or family’s income, occupational status, locality, and educational level [12]. Low SES is disproportionately associated with increased rates of overweight and obesity in high-income countries [5, 13], and individuals experience higher levels of obesity-related diseases, especially cardiovascular disease [14]. A meta-analysis demonstrated that those living in a low SES neighbourhood had a 30% increased risk of being overweight (pooled *OR* 1.30, 95% *CI*; 1.16–1.47, *p* < 0.001) and a 45% increased risk of being obese (pooled *OR* 1.45, 95% *CI*; 1.21–1.74, *p* < 0.001) compared with individuals living in high SES neighbourhoods [15]. People living in deprived areas are more likely to have unhealthy lifestyle behaviours (e.g. smoking, increased alcohol consumption) and lower healthy behaviours (e.g. physical activity, healthy diet) compared to less deprived areas [16]. It has been suggested that the built environment that someone lives in directly influences their lifestyle behaviours. Indeed, areas of higher deprivation have a higher concentration of features that are harmful to health, such as more fast food outlets and limited physical activity opportunities, termed the obesogenic environment [17, 18]. Good quality evidence-based interventions are lacking for people living with overweight and obesity from lower SES. Low SES individuals have worse outcomes and higher dropout rates in health promotion programmes compared to individuals from higher SES [19, 20] due to financial costs of travelling to face-to-face sessions [21], childcare issues and taking time out of work [22] as well as programmes not addressing the structural barriers faced by those with a low SES [23]. These barriers need to be considered in the development of health promotion interventions.

Electronic health (eHealth) is one approach that aims to overcome these barriers, allowing participants to access weight management programmes at times and locations that suit the individual [24]. While results of eHealth interventions have been inconsistent, a recent meta-analysis of 9 pooled studies demonstrated that eHealth weight loss interventions resulted in modest weight loss compared with no treatment (mean difference: −2.70 kg (95% *CI*: −3.33 to −2.08kg); *p* < 0.001); however, their analysis did not account for SES [25]. eHealth interventions vary but utilise technology to provide remote health care to individuals. This may be through the mode of delivery such as computer or mobile phone, utilising websites/web applications, mobile and/or social media applications, email or SMS text messaging [26, 27]. It offers the potential for a wide-reaching, low-cost and efficacious intervention, while also addressing specific barriers associated with people with low SES [22, 28]. But it is unknown whether any eHealth approaches exist for people living with low SES especially as a digital divide still exists where people with low SES are less likely to be able to access eHealth [24]. Furthermore, it is also unknown whether any eHealth interventions have any effect on overweight or obesity in people with low SES.

### Objectives

A systematic review was therefore undertaken to identify eHealth weight management interventions for people living with overweight and obesity from a low SES. The primary aim was to establish what eHealth weight management interventions exist for people with overweight and obesity from a low SES. The secondary aim was to determine the efficacy of interventions in facilitating weight loss and physical activity and fitness improvements in people living with overweight and obesity from a low SES background.

## Method

### Protocol and registration

The protocol for this systematic review was registered with the International Prospective Register of Systematic Reviews (PROSPERO, registration number: CRD42021243973). This systematic review was conducted in accordance with the PRISMA (Preferred Reporting Items for Systematic Reviews and Meta-Analysis) statement guidelines [29] (Supplementary material) and follows a predetermined published protocol [30].

### Eligibility criteria

This review included studies of eHealth weight management interventions in adults over the age of 18 living with overweight or obesity from a low SES background. The PICOS (Population, Intervention, Comparison, Outcomes, and Study design) framework was used to structure the eligibility criteria [31]. Retrieved work was reviewed if it met the inclusion criteria, or was otherwise excluded as per our published protocol [30]. Studies were excluded if they involved bariatric surgery or pharmacology-only interventions, and did not include or report on participants based on SES. Physiological measures were added as an inclusion criterion, and non-eHealth interventions (i.e. face-to-face components) were also added as an exclusion criterion for completeness (Table 1).

**Table 1:** Study eligibility criteria using the PICOS criteria

PICOS	Inclusion	Exclusion
Population	• Adults ≥ 18 years old with BMI > 25 kg/m^2^ • Low SES	• Pregnancy or postpartum (within 3 months) • Any SES other than low SES
Intervention	• Weight management intervention delivered using eHealth technology	• Bariatric surgery • Medication-only interventions • Face-to-face components
Comparator	• N/A^a^	• N/A
Outcome	• Weight (kg), BMI (kg/m^2^) and/or percentage weight change • A range of anthropometric, physiological and physical activity/fitness measures	• N/A
Study design	• Experimental studies • Observational studies • Case studies/series	• Reviews • Secondary analysis

^a^*N/A* Not applicable

### Population

Studies were included if participants were adults over the age of 18, had a BMI greater than 25 kg/m^−2^ and were from a low SES background. Studies were required to explicitly state their criteria of low SES to be included, or outcomes had been reported by SES. Low SES was defined through multiple constructs, including, but not limited to, low income, low educational level, low occupational status or a combination of these [12] (Table 2).

**Table 2:** Outline of domains that relate to socioeconomic status

Domain	Explanation
Income	The earnings received through employment by an individual or family, typically compared against the nation’s average earnings [32]
Education	An indicator for knowledge and educational attainment, generally measured using the individuals highest level of schooling achieved, such as primary, secondary and tertiary education [19]
Occupational status	Involves specific aspects related to the job role itself such as power, income and educational requirements as well as the physical or hazardous demands related to that job [33]

### Intervention types

We included studies that deployed weight management protocols designed to have an effect on weight loss or maintenance, increase in physical fitness and/or physical activity. Interventions involved one or more of the weight management domains as outlined by NICE [34] including diet and nutrition advice/education, physical activity and behaviour change techniques. Eligible studies delivered their interventions via eHealth inclusive of web-based, mobile applications, text, social media or other related modalities. Bariatric surgery and medicine-only trials were excluded, as well as those that had any face-to-face contact.

### Comparator

Studies with or without a control group were considered for eligibility, and no limitation was placed on the control group.

### Outcomes

The primary outcome domains were weight, weight change and BMI. Secondary outcome domains included anthropometric, physiological, fitness or physical activity measures. Outcome domains within included studies were assessed at baseline and at any reported follow-up time point(s) upon completion of the intervention. Studies with multiple time points were reported and the maximum follow-up time selected.

### Study design

Experimental and observational cohort studies that aimed to investigate the efficacy of eHealth weight management interventions that were written in the English language were included. Experimental studies included randomized controlled trials (RCTs), quasi-experimental studies, controlled clinical trials or cluster trials. Quasi-experimental study designs differ from RCTs in that they do not directly manipulate the independent variable, therefore may not include a control group or randomisation [35]. Observational studies comprised of prospective and retrospective comparative cohort studies as well as cross-sectional, case-control or nested case-control studies. A range of study designs were included to identify the breadth of research available. Review articles, secondary analyses and case studies were excluded.

### Search strategy

The systematic literature search was completed in May 2021. The electronic literature search strategy was based on the eligibility criteria using Medical Subject Headings (MeSH) and text words. Electronic databases included MEDLINE, Embase, EmCare and CINAHL. Subject header and free text searches were completed, using Boolean search techniques such as “AND” and “OR”, based on the PICOS framework (Table 1) and previous literature [36]. The detailed search strategy is presented in Supplementary material. Reference lists, grey literature and completed theses were also searched. Databases were searched from their respective inception dates.

### Data collection and analysis

#### Study selection

After the initial search, results were transferred to reference manager software (EndNote X8.0.1, Bld 10444, Clarivate™, London, UK) and duplicates removed. Two authors (R. M. I. and J. S.) independently screened titles and abstracts before full-text articles according to the eligibility criteria, using proprietary systematic review software (Rayyan Systems Inc., Cambridge, MA, USA). Reasons for exclusions were collated, and discrepancies were resolved following discussion and consensus by two authors (R. M. I. and J. S.). If consensus could not be reached, then a third author (GDJ) was available to assess and resolve the discrepancy.

#### Data extraction

An adapted data extraction form was created based on the Cochrane Data Extraction Form for RCTs and non-RCTs [37]. Data included study details (author, year of publication and country), design, participant characteristics (sample size, baseline characteristics including age, ethnicity and SES), interventions and all outcomes post-intervention and any follow-up time points. The same two authors independently extracted data using the form, with any discrepancies settled following an assessment by a third author (G. D. J.).

#### Quality

The same two authors independently assessed the risk of bias of included publications using the Joanna Briggs Institute (JBI) Critical Appraisal Tool, Checklist for Randomised Controlled Trials and Checklist for Quasi-Experimental Studies [38]. Each domain within the JBI Checklist is assigned 0 for low risk of bias, 1 for unclear and 2 for high risk of bias. The total score was calculated into a percentage dependent on the individual checklist used. A final rating of > 50% was deemed as high risk.

#### Data analysis

While meta-analyses of standardised post-intervention outcomes and any similarly-timed follow-ups were intended, the heterogeneity of studies was evaluated and was found to be high for aims, outcome time points and intervention components. Therefore, meta-analyses were not performed, and a narrative synthesis was performed on work included for review [39].

## Results

### Study selection

**Fig. 1:**
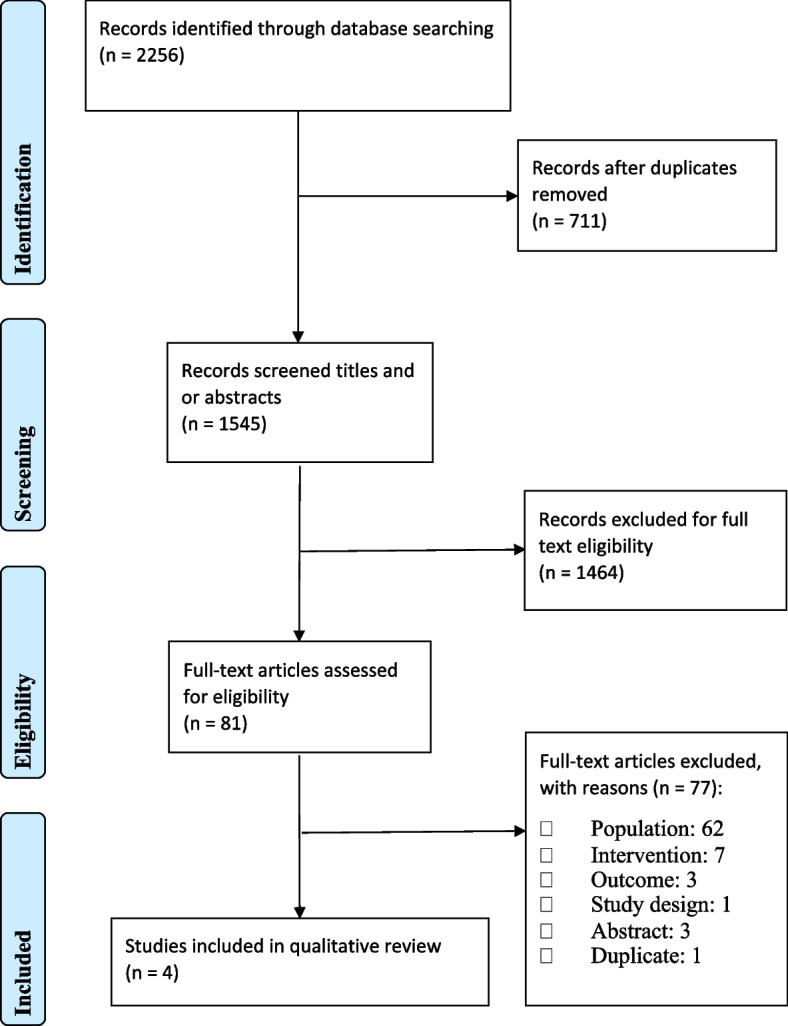
PRISMA flowchart

In total, 2256 studies were identified. After 711 duplicates were removed, 1545 articles remained for title and abstract review, and 1464 were excluded for not meeting the inclusion criteria. Therefore, 81 articles were subjected to full-text assessment of their eligibility. In 62 articles, the population did not include overweight or obese participants from a low SES, 7 did not include eHealth and/or had elements of face-to-face interaction as part of the intervention, 3 did not include the eligible primary or secondary outcomes, 1 did not meet the study design criteria, 3 were not full-text articles and there was 1 duplicate. Four studies were therefore eligible for full-narrative review (Fig. 1).

### Study characteristics

There were 373 participants in total (Table 4). Participants were predominately female (99%); in 3 studies, all participants were females [40, 41, 42] and represented 95% of participants in the remaining article [43]. Ethnicity varied. All participants identified as African American in 1 article [41], as Latinas in another [40] and as multiple ethnicities in the remaining articles [42, 43]. All studies were conducted in the USA. Designs included 3 quasi-experimental [40, 42, 43] and 1 randomised controlled trial [41]. Intervention aims varied between studies; 2 focused on weight loss [42, 43], 1 on weight maintenance [41] and 1 on increasing physical activity [40]. The definition of SES varied. Two articles adopted a percentage of income compared to the national poverty line approach [41, 43], 1 used eligibility for a national nutritional benefits scheme [42] and SES was operationalised as a combination of income, education and employment in the remaining article [40]. The duration of intervention ranged from 1 month [40] to 12 months [41]. Reported attrition rates varied across the included studies from as low as 5% [41], to 12.5% [40], 15% [43] and up to as much as 48.5% [42].

### Risk of bias

All studies had a low risk of bias (Table 3). In the three quasi-experimental studies [40, 42, 43], there were no differences in terms of care received, all included multiple measurements of outcomes (pre and post intervention), outcomes were measured in a standardised way and assessed using appropriate statistical analyses, but none included a control group. In the only RCT [41], participants were randomized, between-group characteristics were insignificantly different at baseline, they were treated identically except for the intervention, follow-up was complete and appropriate statistical analyses were deployed; however, neither assessors nor participants were blinded to the treatment assignment.

**Table 3:** Quality assessment scores of included studies

Study	Study design	Critical appraisal tool	Score
Benitez et al. [40]	Quasi experimental	JBI Checklist for Quasi-Experimental Studies	2/16
Bennett et al. [41]	RCT	JBI Checklist for Randomized Controlled Trials	8/26
Cavallo et al. [43]	Quasi experimental	JBI Checklist for Quasi-Experimental Studies	2/16
Griffin et al. [42]	Quasi experimental	JBI Checklist for Quasi-Experimental Studies	2/16

### Intervention components

The components of interventions included in this review varied considerably and included an interactive website [40], interactive voice response and monthly telephone calls [41], social media [43], and text messages and eNewsletters [42] (Table 4). Benitez et al. [40] conducted a 1-month intervention providing access to a culturally and linguistically adapted, theory-driven website promoting physical activity. In contrast, Bennett et al. [41] conducted an intervention known as the SHAPE programme where participants were assigned behaviour change goals by a computer algorithm from a library of goals (such as no sugar-sweetened beverages, no fast food and increase fruit and vegetable intake) at baseline and 6 months, as well as monthly telephone calls with a dietitian. Cavallo et al. [43] used a different approach called the INSHAPE CLE intervention. Here, access to a private social media group was provided, with daily online posts focusing on healthy eating advice using different themes such as Education Only, Recipes, Testimonials/Goal Setting, “Ask a Dietitian” and Competitions. Finally, Griffin et al. [42] developed a simple approach called the MyQuest intervention utilising 2 to 3 daily text messages and eNewsletters.

**Table 4:** Characteristic of eHealth weight management studies in low SES adults

Study			Population					Intervention				
Author (year), country	Study design	Setting	*n*	Mean age	Sex % F	Ethnicity	SES domain (s)	Duration/follow-up	Type of technology	Theory	Behaviour aims/ target	Component(s)
Benitez et al. (2015) [40], USA	Quasi exp.	Community	24	35.2	100	Latinas	Combination	1 month/none	Website	SCT TTM	Promote PA	1). Personalised physical activity counselling messages and strategies 2). Access to culturally adapted exercise videos 3). Provided with pedometers 4). Guest access to website
Bennett et al. (2013) [41], USA	RCT	Community	185	35.4	100	African-American	Low income	12 months/18 months	IVR	Self-efficacy theory	Improve well-being maintain weight	Intervention 1). Obesogenic behaviour change goals 2). Self-monitoring via IVR phone calls 3). Tailored skills training materials 4). 12 interpersonal counselling calls 5). 12-month gym membership Control 1). General wellness newsletters every 6 months
Cavallo et al. (2021) [43], USA	Quasi exp.	Community	55	46.4	95	White = 23.6% Black or African American = 69.1% More than one race = 5.9%	Low income	3 months/none	Social media	NR	Weight loss	1). Access to a private social media group — ~3 daily posts by moderator 2). Provided with a Fitbit 3). Monetary incentive
Griffin et al. (2020) [42], USA	Quasi exp.	Community	109	36.1	100	White = 43.1%, African American = 54.1%	Low income	3 months/none	Text messaging eNewsletter	SCT	Increase PA Weight loss	1). Prescribed 1600 kcal/day meal plan 2). Daily text messages including goal setting, healthy eating and PA reminders 3). Weekly eNewsletters 4). Provided with a pedometer
		Total:	373	38.3	98.8							

*IVR*, Interactive voice response, *n* sample size; *NR* not reported, *PA*, Physical activity, *SCT* Social cognitive theory, *TTM* Transtheoretical model, *RCT* Randomised controlled trial

### Weight loss and maintenance effects

Two studies aimed to achieve weight loss [42, 43], and 1 aimed to maintain weight [41]. All reported a significant weight loss at the end of the intervention [41–43] with one observing significant weight loss at 18-month follow-up [41] (Table 5). Mean (±SD) body weight loss ranged from 1.07 (3.96) kg to 1.81 (5.76) kg. Cavallo et al. [43] observed that participants lost ≥ 5% of baseline body weight in 16% of participants, while Griffin et al. [42] observed it in 32% of participants and ≥ 10% in 5% of participants.

**Table 5:** Outcomes of eHealth weight management interventions in low SES adults

Study			Results			
Author (year)	Attrition	Time points	Weight (kg) (Mean (±SD/95% *CI*)	BMI (kg/m^2^) (95% *CI*)	Physical activity measure	Physical activity
Benitez et al. (2015) [40]	12.5%	Baseline 1 month	Not reported	NR	Seven- Day Physical Activity Recall (moderate to vigorous physical activity)	Pre: 12.5 (0–120) min/week Post: 67.5 (0–510) min/week Change: +55 min/week
Bennett et al. (2013) [41]	5%	Baseline 12 months 18 months	12 months IG: −1. 0 (0.5) CG: 0.5 (0.5) Mean difference: −1.4 (−2.8 to −0.1) 18 months IG: −0.9 (0.6) CG: 0.8 (0.6) Mean difference: −1.7 (−3.3 to −0.2)	12 months IG: −0.3 (0.2) CG: 0.3 (0.2) Mean difference: −0.6 (−1.1 to −0.1) 18 months IG: −0.2 (0.2) CG: 0.4 (0.2) Mean difference: −0.6 (−1.2 to −0.1)	N/A	N/A
Cavallo et al. (2021) [43]	15%	Baseline 3 months	Pre: 95.38 (12.33) Post: 94.31 (13.21) Change: −1.07 (−2.14 to 0.0)	NR	N/A	N/A
Griffin et al. (2020) [42]	48.5%	Baseline 3 months	Pre: 92.35 Post: 89.9 Change: −1.81 (5.76)	NR	Pedometer	Pre: 6819 steps/day Post: 8980 steps/day Change: +1689 (689) steps/day

*CG*, control group, *IG*, Intervention group, *NR*, Not reported, *N/A*, Not applicable

### Physical activity and fitness effects

Two studies aimed to increase physical activity [40, 42]. Both observed a statistically significant increase in physical activity, although methods of measurements differed. Benitez et al. [40] reported a median (range) increase in moderate to vigorous physical activity using the 7-Day Physical Activity Recall from 12.5 (0–120) to 67.5 min (0–510) (*p* = < 0.05). In contrast, Griffin et al. [42] reported physical activity using pedometers to measure daily steps. There was a significant mean (±SD) difference in daily steps between baseline (6819) and post intervention (8980) of 1689 (±689) steps (*p* = 0.19). No studies reported any effects on physical fitness.

### Anthropometry and physiological effects

Only 1 study [41] reported outcomes for anthropometric and physiological measures. No significant differences between intervention and control groups were found in waist circumference, blood pressure, blood pressure control, glucose or lipid levels at any time point.

## Discussion

### Main findings

To the authors’ knowledge, this is the first attempt to systematically review the literature of weight management interventions using eHealth specifically in people from a low SES background and living with overweight and obesity. It is important because low SES individuals are disproportionately affected by overweight and obesity [13]. The main findings are that eHealth interventions specifically designed for low SES groups are scarce with only 4 low risk-of-bias studies meeting our inclusion criteria, comprising a total of 373 participants. eHealth interventions aiming to reduce/maintain weight or increase physical activity varied. They included interactive websites or voice responses, periodic communication and discourse via telephone, social media, text messaging or eNewsletters. All studies reported a significant effect of their respective eHealth interventions on weight loss. Generalisations should be made with caution however as the review revealed only USA-centric studies with predominantly female participants and sample sizes were small to modest (ranging between *n* = 24 and *n* = 185). Given that SES spectra are not invariant across nation states nor equally distributed between biological sex [44], and overweight and obesity affect males more than females in the UK [6], future eHealth studies specific to the UK and that include both sexes are required.

### Effect on weight loss

Intervention duration was relatively short (1–3 months, with one exception of 12 months and follow-up at 18 months), yet all interventions demonstrated statistically significant weight loss during the intervention. In the longer intervention, the effect was sustained at 18 months [41]. There was a significant effect of interventions on physical activity which improved at 3 months in two articles [40, 42]. Despite the sample sizes being modest, these findings are welcome and collectively supports the premise that eHealth interventions are a successful approach for people with low SES. Our findings are in keeping with an earlier narrative systematic review (6 studies, *n* = 4899 [36]). It observed that eHealth weight management interventions had a positive effect on weight loss in participants who identified as being part of an ethnic minority group. Given that ethnic minorities are also associated with higher risk of deprivation and obesity [45], there is further evidence eHealth is an efficacious approach for vulnerable groups within the general population.

Although in our review we found interventions led to statistically significant weight loss, these findings need to be interpreted with respect to a *clinically* significant weight loss. According to UK clinical guidance, 3–5% body weight loss is associated with clinically meaningful health benefits [34], and aiming for 30% of participants achieving 5% weight loss is a desirable service outcome [46]. Two studies reviewed [42, 43] reported 16% and 32% of participants achieved ≥ 5% of body weight loss respectively, meaning a minority of low SES participants achieved a *clinically* significant weight, and one did not meet the UK national guidance. There is a need therefore to develop successful interventions to achieve clinically meaningful weight loss in a greater proportion of participants.

### Effect on physical activity

Economic, social and political factors influence and, to some degree, drive the amount of physical activity and exercise completed at the population level, seeing as uptake of global recommendations (e.g. [47]) remains low [48]. No study reviewed assessed the effect on physical fitness which is presumably because physical fitness is defined as a subset of physical activity [49]. It might also be due to the recognition of attitudinal differences towards exercise compared with physical activity in people with long-term conditions [50, 51]. Irrespectively, physical activity increased significantly as an effect of eHealth programmes in two studies included in the current review [40, 42]. Since optimising physical activity and exercise as a behaviour change is desirable to support and maintain weight loss and reduces the risk of noncommunicable diseases [52], evidencing eHealth’s effectiveness in increasing physical activity for low SES participants supports targeting physical activity in the design of interventions for this group.

People with low SES face specific barriers to sustained physical activity changes such as the cost of gym membership, perceived neighbourhood safety and availability of green spaces to be physically active in [22, 53]. Efforts to modulate these barriers should be included in the design of interventions. Improving self-efficacy is a positive predictor of increasing physical activity in low SES groups [54]. So, it was welcome that self-efficacy was included within the eHealth interventions in the reviewed studies by provision of tailored physical activity feedback, pedometer self-monitoring and setting physical activity goals [40, 42]. But it was disappointing that neither were able to report whether physical activity changes were sustained after 1-month [40] and 3-month [42] intervention periods. A previous systematic review and meta-analysis with low-income participants identified that while interventions resulted in a small but significant increase in physical activity levels, the effect was modest compared to interventions involving the general population, and it was not maintained at 6 months [55]. Furthermore, interventions were not limited to solely eHealth, and some included studies containing face-to-face components. Evidence supporting the relative effect of eHealth on physical activity levels in low compared to higher SES participants, and whether any increases are sustained, therefore remains elusive.

### eHealth interventions and media

The reviewed studies supported behaviour change through increasing self-monitoring behaviours (e.g. interactive voice response (IVR) and text messages) and information provision (e.g. social media posts and eNewsletters). Three studies provided equipment to support self-monitoring of physical activity [40, 42, 43]. One provided access to a gym with reimbursement of travel costs for follow-up visits [41]. Weight loss outcomes in this study were compelling and sustained at 18 months which suggests that providing financial support could be a significant behavioural modifier given that absorbing travel costs is a specific barrier identified in low SES groups. Access to gyms, walking groups and community involvement are effective strategies to prevent weight gain in low SES groups [23]. Thus, it is no surprise that interventions that consider environmental, social, economic and/or structural issues are more likely to improve outcomes across SES. In the development of future interventions, clinicians, researchers and funders have an obligation to consider factors associated with low SES, such as insufficient financial agency to purchase interventions and self-monitoring equipment. At a national level, financial support for sustained public health could be provided as part of welfare systems. There is debate whether the advanced welfare tax burden that egalitarian societies sustain offsets health inequalities due to socioeconomic status compared to more neoliberal welfare states [56]. Our belief is that the investigations into the causes for health inequalities should continue and are welcome because they will provide testable theories that can explain, for example, how physical activity improvements due to eHealth interventions wane differently depending on SES and why. These may well indicate that provision of sustained financial support programmes for eHealth as a public health intervention is indicated for subgroups of society, and if so, programmes should be duly scrutinised for their cost-effectiveness.

eHealth has the potential to improve health at local, national and international levels by using the developing technology effectively. Counterintuitively though, an expanding eHealth landscape could widen social health inequalities because not all individuals are able to use eHealth well due to inequity and inequality in environmental factors, access, cost and utilisation [24]. Inequality exists in the dissemination of intervention results to the public too. Due respect to the spectrum of health literacy in the public to whose behaviours the results are aimed at modifying is not always made. What’s more, our results identify the scarcity of studies that included low SES participants. This potential bias is vexing because individuals with low SES are at a greater risk of social health inequalities. There is therefore a clear need to focus eHealth interventions tailored to this group.

The delivery method of eHealth should be an important factor when developing interventions due to differing utilisation of technology across SES. eHealth that is not accessible, easy to use and/or targeted to the population may further the digital divide [57]. Using smartphones as the only access to the internet is high among low-income groups [58]. This means the use of mobile technology and applications may be more appropriate and acceptable in this population. While the interventions revealed in this review could all have been practically accessed using a smartphone, only one study was specifically designed for smartphone use via direct text messaging — a modality which incidentally caused the largest mean change in body weight loss [42]. Three studies did not specifically describe the use of smartphone use or accessibility despite the potential this has in this population. Utilising or adapting eHealth for smartphone compatibility should be supported because it is a strong candidate to improve the efficacy of interventions while minimising health inequalities among low SES groups [59].

### Uptake and attrition

Uptake and attrition are key challenges in investigating weight management interventions in individuals with low SES due to the complex behaviour change required [60]. Attrition rates were generally low in included studies compared to traditional weight management interventions where attrition rates can be up to 80% [60]. Bennett et al. [41] reported the lowest attrition rate (5%), presumably due to the strict exclusion criteria removing any participants that were suspected of being “uninterested”. Griffin et al. [42] observed the highest attrition rate (48.5%) among participants who identified as African American and participants with the lowest education and incomes. This suggests there may be sub-groups within low SES along ethnicity, education and income demographics and presumably their intersections. Understanding the reasons for the demographic differences in completing programmes is an important area for further research.

Engaging sub-groups in the development of interventions, and understanding their specific needs, is likely to improve retention of participants and outcomes. Barriers to participation in interventional studies are well documented [61]. In addition to experiencing significant time demands to attend and travel to study appointments, people with low SES report mistrust of, and poor communication with, physicians and nurses [62], and it would be interesting to see if similar barriers exist for people with low SES in their interactions with other health professionals for instance exercise physiologists and prescribers or nutritionists. Irrespectively, eHealth has the potential to overcome some of these barriers because it can offset time and costs and provides autonomy in selecting to participate at convenient times.

### Strengths and limitations

This systematic review was registered with an international systematic review register which is one of its strengths. It has been written following the PRISMA guidelines [29], and the protocol has been previously published [30]. We do however acknowledge some limitations. The review only included adults. Given that the burden of overweight and obesity is growing, there is a need to identify how eHealth can be utilised across the lifespan including younger populations who have different digital habits. This review identified only a small number of eligible studies. This was mainly due to many studies not specifying the SES criteria used or involving participants across the SES spectrum. We specifically wanted interventions that solely targeted people from low SES as we defined it. The complex nature of SES and its varying constructs and domains mean a standardised definition of low SES remains elusive, and we acknowledge that our definition may not have yielded all relevant studies. It is therefore possible that studies were not identified within our search strategy that analysed participants in subgroupings that might have satisfied our inclusion criteria.

## Conclusions

In summary, there is a small amount of evidence with low risk of bias within the literature supporting eHealth interventions for weight management in people with low SES — a group of society who are often under represented within research. This systematic review has demonstrated that eHealth weight management interventions can lead to short-term weight loss and increases in physical activity in people with low SES. It must be recognised, however, that this interpretation is based on a small number of studies with small to modest sample sizes, as well as generally low-quality study designs. Hence, more thoroughly designed experimental studies are indicated. eHealth has the potential to deliver evidence-based interventions with high reach and low cost, but intervention designers and funders should be mindful of widening social health inequalities if there are members of society who are inadvertently subjected to discrimination based on their ability to access eHealth. Our findings, in contrast, have shown that it is feasible for people with low SES to utilise eHealth. This review therefore supports the idea of promoting of eHealth interventions to support people living with overweight and obesity in low SES groups with specific consideration of the delivery components (e.g. smart phones, mobile applications and social media), the structural factors associated with SES, the specification and tailoring of interventions and the assessment of sustained behaviour change.

## Supplementary Information

**Additional file 1.** PRISMA_2020_checklist. Completed PRISMA checklist for systematic review’s and meta-analyses.

**Additional file 2.** Search Strategy- eHealth interventions for weight management in adults with low socio-economic status. Example search strategies completed on MEDLINE and CINAHL databases.

**Additional file 3.** Excluded Studies. A list of articles that may have appeared to meet the inclusion criteria, but which were excluded following full text review.

## Data Availability

N/A.

## Abbreviations

BMI: Body mass index
CI: Confidence interval
eHealth: Electronic health
IVR: Interactive voice response
JBI: Joanna Briggs Institute
MeSH: Medical Subject Headings
NICE: National Institute for Health and Care Excellence
OR: Odds ratio
PRISMA: Preferred Reporting Items for Systematic Reviews and Meta-Analyses
PROSPERO: International Prospective Register of Systematic reviews
RCT: Randomised controlled trial
SES: Socioeconomic status
SMS: Short message service
WHO: World Health Organization
