# Supplementary material for: Effectiveness of eHealth weight management interventions in overweight and obese adults from low socioeconomic groups: a systematic review
Source: Syst Rev. 2023 Mar 30;12:59. doi: 10.1186/s13643-023-02207-3 (PMC10061957; doi:10.1186/s13643-023-02207-3)
Supplement: Supplementary file 2 — Additional file 2. Search Strategy- eHealth interventions for weight management in adults with low socio-economic status. Example search strategies completed on MEDLINE and CINAHL databases. [file 13643_2023_2207_MOESM2_ESM.pdf]

Database Search Overview

Database	Date searched	Results
CINAHL Plus with Full Text (Ebsco)	06/05/2021	218
Embase (Elsevier)	06/05/2021	989
EMCARE (Elievier)	06/05/2021	425
Ovid MEDLINE(R) and Epub Ahead of Print, In-Process & Other Non-Indexed Citations, Daily and Versions(R) 1946 to May 05, 2021	06/05/2021	624
**Total**		**2256**
**After duplicates removed**		**1545**

We searched the databases listed above on 6th May 2021. Records from each database were exported to EndNote. Duplicates were removed using hand searching and the "Find Duplicates" function within EndNote. Unique results were uploaded to Rayyan. Example search strategies from each of the bibliographic databases are available below.

Database Example:
Ovid MEDLINE(R), and Epub Ahead of Print, In-Process, In-Data-Review & Other Non-Indexed Citations and Daily <1946 to May 05, 2021>

#	Query	Results from 6 May 2021
1	exp Obesity/	224,286
2	exp Overweight/	231,043
3	exp Adult/	7,442,878
4	exp Social Class/ or exp Socioeconomic Factors/ or exp Health Status Disparities/	474,908
5	exp Health Status Disparities/ or exp Health Services Accessibility/	131,230
6	exp Poverty/	45,299
7	exp Income/	65,099
8	exp Educational Status/	53,562
9	exp Employment/	90,034
10	Obesity.mp. [mp=title, abstract, original title, name of substance word, subject heading word, floating sub-heading word, keyword heading word, organism supplementary concept word, protocol supplementary concept word, rare disease supplementary concept word, unique identifier, synonyms]	346,052
11	obese.mp. [mp=title, abstract, original title, name of substance word, subject heading word, floating sub-heading word, keyword heading word, organism	135,462

	supplementary concept word, protocol supplementary concept word, rare disease supplementary concept word, unique identifier, synonyms]	
12	overweight.mp. [mp=title, abstract, original title, name of substance word, subject heading word, floating sub-heading word, keyword heading word, organism supplementary concept word, protocol supplementary concept word, rare disease supplementary concept word, unique identifier, synonyms]	79,302
13	adult.mp. [mp=title, abstract, original title, name of substance word, subject heading word, floating sub-heading word, keyword heading word, organism supplementary concept word, protocol supplementary concept word, rare disease supplementary concept word, unique identifier, synonyms]	5,789,631
14	"Social disparit$".mp. [mp=title, abstract, original title, name of substance word, subject heading word, floating sub-heading word, keyword heading word, organism supplementary concept word, protocol supplementary concept word, rare disease supplementary concept word, unique identifier, synonyms]	636
15	"Social inequalit$".mp. [mp=title, abstract, original title, name of substance word, subject heading word, floating sub-heading word, keyword heading word, organism supplementary concept word, protocol supplementary concept word, rare disease supplementary concept word, unique identifier, synonyms]	4,047
16	"Social inequity".mp. [mp=title, abstract, original title, name of substance word, subject heading word, floating sub-heading word, keyword heading word, organism supplementary concept word, protocol supplementary concept word, rare disease supplementary concept word, unique identifier, synonyms]	166
17	"Economic disparity".mp. [mp=title, abstract, original title, name of substance word, subject heading word, floating sub-heading word, keyword heading word, organism supplementary concept word, protocol supplementary concept word, rare disease supplementary concept word, unique identifier, synonyms]	99
18	"Economic inequality".mp. [mp=title, abstract, original title, name of substance word, subject heading word, floating sub-heading word, keyword heading word, organism supplementary concept word, protocol supplementary concept word, rare disease supplementary concept word, unique identifier, synonyms]	579
19	"Economic inequity".mp. [mp=title, abstract, original title, name of substance word, subject heading word, floating sub-heading word, keyword heading word, organism supplementary concept word, protocol supplementary concept word, rare disease supplementary concept word, unique identifier, synonyms]	43
20	"Socio-economic disparity".mp. [mp=title, abstract, original title, name of substance word, subject heading word, floating sub-heading word, keyword heading word, organism supplementary concept word, protocol supplementary concept word, rare disease supplementary concept word, unique identifier, synonyms]	31
21	"Socioeconomic disparity".mp. [mp=title, abstract, original title, name of substance word, subject heading word, floating sub-heading word, keyword heading word, organism supplementary concept word, protocol supplementary concept word, rare disease supplementary concept word, unique identifier, synonyms]	168
22	"Socioeconomic inequality".mp. [mp=title, abstract, original title, name of substance word, subject heading word, floating sub-heading word, keyword heading word, organism supplementary concept word, protocol supplementary concept word, rare disease supplementary concept word, unique identifier, synonyms]	650
23	"Socio-economic inequality".mp. [mp=title, abstract, original title, name of substance word, subject heading word, floating sub-heading word, keyword heading word,	141

	organism supplementary concept word, protocol supplementary concept word, rare disease supplementary concept word, unique identifier, synonyms]	
24	"Socioeconomic inequity".mp. [mp=title, abstract, original title, name of substance word, subject heading word, floating sub-heading word, keyword heading word, organism supplementary concept word, protocol supplementary concept word, rare disease supplementary concept word, unique identifier, synonyms]	58
25	"Socio-economic inequity".mp. [mp=title, abstract, original title, name of substance word, subject heading word, floating sub-heading word, keyword heading word, organism supplementary concept word, protocol supplementary concept word, rare disease supplementary concept word, unique identifier, synonyms]	18
26	"Low income".mp. [mp=title, abstract, original title, name of substance word, subject heading word, floating sub-heading word, keyword heading word, organism supplementary concept word, protocol supplementary concept word, rare disease supplementary concept word, unique identifier, synonyms]	38,756
27	"Low education".mp. [mp=title, abstract, original title, name of substance word, subject heading word, floating sub-heading word, keyword heading word, organism supplementary concept word, protocol supplementary concept word, rare disease supplementary concept word, unique identifier, synonyms]	4,293
28	Employment.mp. [mp=title, abstract, original title, name of substance word, subject heading word, floating sub-heading word, keyword heading word, organism supplementary concept word, protocol supplementary concept word, rare disease supplementary concept word, unique identifier, synonyms]	93,387
29	1 or 2 or 10 or 11 or 12	393,151
30	3 or 13	7,917,894
31	4 or 5 or 6 or 7 or 8 or 9 or 14 or 15 or 16 or 17 or 18 or 19 or 20 or 21 or 22 or 23 or 24 or 25 or 26 or 27 or 28	627,216
32	exp Telemedicine/	34,082
33	"electronic health".mp.	34,562
34	telemedicine.mp.	35,212
35	telehealth.mp.	7,344
36	mhealth.mp.	5,842
37	m-health.mp.	648
38	"mobile health".mp.	8,990
39	"interactive media".mp.	146
40	exp Telephone/	29,294
41	telephone.mp. [mp=title, abstract, original title, name of substance word, subject heading word, floating sub-heading word, keyword heading word, organism supplementary concept word, protocol supplementary concept word, rare disease supplementary concept word, unique identifier, synonyms]	65,271
42	telephone-based.mp.	1,925
43	phone-based.mp.	1,252
44	exp Internet Access/ or exp Internet/	84,525
45	internet.mp.	110,792
46	internet-based.mp.	9,577
47	exp Web Browser/	1,243
48	web.mp.	131,549
49	web-based.mp.	33,806

50	exp Information Dissemination/ or exp Social Media/	26,677
51	website.mp.	19,571
52	website-based.mp.	100
53	exp Electronic Mail/	2,763
54	e-mail.mp.	7,255
55	exp Telecommunications/	104,449
56	"electronic mail".mp.	3,460
57	exp Computers/	80,018
58	computers.mp.	73,398
59	computer.mp.	725,533
60	computer-based.mp.	14,357
61	exp Wireless Technology/	3,846
62	wireless.mp.	16,807
63	exp Cell Phone/	17,091
64	"mobile phone".mp.	7,823
65	"cell phone".mp.	10,377
66	"cellular phone".mp.	565
67	exp Smartphone/	5,741
68	smartphone.mp.	13,667
69	exp Computers, Handheld/	9,391
70	"mobile device".mp.	1,339
71	"personal digital assistant".mp.	580
72	pda.mp.	13,097
73	"interactive voice response".mp.	851
74	ivr.mp.	1,569
75	exp Text Messaging/	3,396
76	"text message".mp.	1,817
77	"text messaging".mp.	4,680
78	SMS.mp.	6,547
79	bluetooth.mp.	1,371
80	chat.mp.	8,641
81	"chat room".mp.	140
82	"instant message".mp.	22
83	IM.mp.	117,272
84	exp Social Media/	9,910
85	twitter.mp.	4,020
86	tweet.mp.	574
87	exp Blogging/	1,016
88	blog.mp.	872
89	exp Social Networking/	4,498
90	"social network".mp.	10,338
91	tailored.mp.	67,223
92	automated.mp.	146,962
93	"individualized programme".mp.	18

94	"individualised programme".mp.	17
95	exp Remote Consultation/ or remote.mp.	82,708
96	self-monitoring.mp.	13,501
97	exp Feedback/ or feedback.mp.	164,916
98	prompt.mp.	68,323
99	reminder.mp.	10,349
100	32 or 33 or 34 or 35 or 36 or 37 or 38 or 39 or 40 or 41 or 42 or 43 or 44 or 45 or 46 or 47 or 48 or 49 or 50 or 51 or 52 or 53 or 54 or 55 or 56 or 57 or 58 or 59 or 60 or 61 or 62 or 63 or 64 or 65 or 66 or 67 or 68 or 69 or 70 or 71 or 72 or 73 or 74 or 75 or 76 or 77 or 78 or 79 or 80 or 81 or 82 or 83 or 84 or 85 or 86 or 87 or 88 or 89 or 90 or 91 or 92 or 93 or 94 or 95 or 96 or 97 or 98 or 99	1,744,879
101	exp Weight Loss/	43,832
102	"weight loss".mp.	104,711
103	"weight reduction".mp.	11,824
104	exp Weight Reduction Programs/ or "weight reduction program*".mp.	3,193
105	"weight loss maintenance".mp.	928
106	exp Weight Gain/ or "weight gain prevention".mp.	33,062
107	"obesity trials".mp.	25
108	"obesity reduction".mp.	195
109	"obesity prevention".mp.	4,278
110	"Body mass index".mp. or exp Body Mass Index/	255,349
111	"BMI".mp.	157,476
112	exp Cardiorespiratory Fitness/	2,072
113	exp Physical Fitness/ or "cardiopulmonary fitness".mp.	32,366
114	"cardiovascular fitness".mp.	1,418
115	VO2max.mp.	9,246
116	"estimated VO2max".mp.	180
117	"predicted VO2peak".mp.	50
118	exp Exercise Tolerance/ or "aerobic capacity".mp.	18,115
119	"physical activity".mp. or exp Exercise/	281,548
120	"physical fitness".mp.	32,928
121	"aerobic fitness".mp.	3,375
122	101 or 102 or 103 or 104 or 105 or 106 or 107 or 108 or 109 or 110 or 111 or 112 or 113 or 114 or 115 or 116 or 117 or 118 or 119 or 120 or 121	686,745
123	"weight loss program*".mp. [mp=title, abstract, original title, name of substance word, subject heading word, floating sub-heading word, keyword heading word, organism supplementary concept word, protocol supplementary concept word, rare disease supplementary concept word, unique identifier, synonyms]	2,448
124	exp Weight Reduction Programs/	2,547
125	diet.mp. or exp Diet, Healthy/ or exp "Diet, Food, and Nutrition"/ or exp Diet/ or exp Diet, Ketogenic/ or exp Diet, High-Protein Low-Carbohydrate/ or exp Diet, Carbohydrate-Restricted/	1,923,039
126	nutrition.mp.	242,809
127	"Physical activity".mp. or exp Exercise/	281,548
128	exercise.mp.	371,854

129	"weight management program*".mp. or exp Weight Reduction Programs/	3,503
130	"weight management intervention".mp.	278
131	exp Weight Loss/ or "weight loss intervention".mp.	44,361
132	exp Health Behavior/ or "behaviour change".mp. or exp Behavior Therapy/	408,345
133	"behaviour change techniques".mp.	644
134	"Low calorie diet".mp. or exp Caloric Restriction/	7,571
135	"very low calorie ketogenic diet".mp.	50
136	123 or 124 or 125 or 126 or 127 or 128 or 129 or 130 or 131 or 132 or 133 or 134 or 135	2,821,267
137	29 and 30 and 31 and 100 and 122 and 136	649
138	137	649
139	limit 137 to (english language and humans)	624

Database Example:
CINAHL Plus with Full Text (Ebsco)

#	Query	Limiters/Expanders	Results
S110	S4 AND S105 AND S106 AND S107 AND S108 AND S109	Expanders - Apply equivalent subjects<br>Search modes - Boolean/Phrase	218
S109	S85 OR S86 OR S87 OR S88 OR S89 OR S90 OR S91 OR S92 OR S93 OR S94 OR S95 OR S96 OR S97 OR S98 OR S99 OR S100 OR S101 OR S102 OR S103 OR S104	Expanders - Apply equivalent subjects<br>Search modes - Boolean/Phrase	260,977
S108	S73 OR S74 OR S75 OR S76 OR S77 OR S78 OR S79 OR S80 OR S81 OR S82 OR S83 OR S84	Expanders - Apply equivalent subjects<br>Search modes - Boolean/Phrase	383,086
S107	S20 OR S21 OR S22 OR S23 OR S24 OR S25 OR S26 OR S27 OR S28 OR S29 OR S30 OR S31 OR S32 OR S33 OR S34 OR S35 OR S36 OR S37 OR S38 OR S39 OR S40 OR S41 OR S42 OR S43 OR S44 OR S45 OR S46 OR S47 OR S48 OR S49 OR S50 OR S51 OR S52 OR S53 OR S54 OR S55 OR S56 OR S57 OR S58 OR S59 OR S60 OR S61 OR S62 OR S63 OR S64 OR S65 OR S66 OR S67 OR	Expanders - Apply equivalent subjects<br>Search modes - Boolean/Phrase	571,502

	S68 OR S69 OR S70 OR S71 OR S72		
S106	S5 OR S6 OR S7 OR S8 OR S9 OR S10 OR S11 OR S12 OR S13 OR S14 OR S15 OR S16 OR S17 OR S18 OR S19	Expanders - Apply equivalent subjects<br>Search modes - Boolean/Phrase	198,728
S105	S1 OR S2 OR S3	Expanders - Apply equivalent subjects<br>Search modes - Boolean/Phrase	145,120
S104	"aerobic fitness"	Expanders - Apply equivalent subjects<br>Search modes - Boolean/Phrase	1,560
S103	"physical fitness"	Expanders - Apply equivalent subjects<br>Search modes - Boolean/Phrase	19,883
S102	"physical activity"	Expanders - Apply equivalent subjects<br>Search modes - Boolean/Phrase	101,171
S101	"aerobic capacity"	Expanders - Apply equivalent subjects<br>Search modes - Boolean/Phrase	3,597
S100	"predicted VO2peak"	Expanders - Apply equivalent subjects<br>Search modes - Boolean/Phrase	19
S99	"estimated VO2max"	Expanders - Apply equivalent subjects<br>Search modes - Boolean/Phrase	73
S98	VO2max	Expanders - Apply equivalent subjects<br>Search modes - Boolean/Phrase	2,704
S97	"cardiovascular fitness"	Expanders - Apply equivalent subjects<br>Search modes - Boolean/Phrase	729
S96	"cardiopulmonary fitness"	Expanders - Apply equivalent subjects<br>Search modes - Boolean/Phrase	153
S95	"cardiorespiratory fitness"	Expanders - Apply equivalent subjects<br>Search modes - Boolean/Phrase	3,280

S94	"obesity prevention"	Expanders - Apply equivalent subjects<br>Search modes - Boolean/Phrase	17,197
S93	"obesity reduction"	Expanders - Apply equivalent subjects<br>Search modes - Boolean/Phrase	87
S92	"obesity trials"	Expanders - Apply equivalent subjects<br>Search modes - Boolean/Phrase	5
S91	"weight gain prevention"	Expanders - Apply equivalent subjects<br>Search modes - Boolean/Phrase	894
S90	"weight loss maintenance"	Expanders - Apply equivalent subjects<br>Search modes - Boolean/Phrase	483
S89	"weight reduction"	Expanders - Apply equivalent subjects<br>Search modes - Boolean/Phrase	14,332
S88	"weight reduction program*"	Expanders - Apply equivalent subjects<br>Search modes - Boolean/Phrase	3,115
S87	(MH "Weight Reduction Programs") OR (MH "Weight Loss")	Expanders - Apply equivalent subjects<br>Search modes - Boolean/Phrase	24,985
S86	BMI	Expanders - Apply equivalent subjects<br>Search modes - Boolean/Phrase	58,892
S85	(MH "Body Mass Index") OR (MH "Fat Free Mass") OR ""Body mass index""	Expanders - Apply equivalent subjects<br>Search modes - Boolean/Phrase	120,760
S84	“very low calorie ketogenic diet”	Expanders - Apply equivalent subjects<br>Search modes - Boolean/Phrase	15
S83	“Low calorie diet”	Expanders - Apply equivalent subjects<br>Search modes - Boolean/Phrase	2,678
S82	“behaviour change techniques”	Expanders - Apply equivalent subjects<br>Search modes - Boolean/Phrase	409

S81	“behaviour change”	Expanders - Apply equivalent subjects<br>Search modes - Boolean/Phrase	3,490
S80	“weight loss intervention”	Expanders - Apply equivalent subjects<br>Search modes - Boolean/Phrase	700
S79	“weight management intervention”	Expanders - Apply equivalent subjects<br>Search modes - Boolean/Phrase	175
S78	“weight management program*”	Expanders - Apply equivalent subjects<br>Search modes - Boolean/Phrase	798
S77	(MH "Exercise") OR "Exercise" OR (MH "Teaching: Prescribed Activity-Exercise (Iowa NIC)") OR (MH "Aerobic Exercises") OR (MH "Exercise Promotion (Iowa NIC)")	Expanders - Apply equivalent subjects<br>Search modes - Boolean/Phrase	177,838
S76	(MH "Physical Activity") OR "“Physical activity”"	Expanders - Apply equivalent subjects<br>Search modes - Boolean/Phrase	102,472
S75	(MH "Nutrition")	Expanders - Apply equivalent subjects<br>Search modes - Boolean/Phrase	28,755
S74	(MH "Diet, Fat-Restricted") OR (MH "Diet, Low Carbohydrate") OR (MH "Restricted Diet") OR "Diet"	Expanders - Apply equivalent subjects<br>Search modes - Boolean/Phrase	142,020
S73	(MH "Weight Reduction Programs") OR (MH "Weight Reduction Assistance (Iowa NIC)") OR (MH "Weight Loss") OR "“weight loss program*”"	Expanders - Apply equivalent subjects<br>Search modes - Boolean/Phrase	25,297
S72	reminder	Expanders - Apply equivalent subjects<br>Search modes - Boolean/Phrase	8,434
S71	prompt	Expanders - Apply equivalent subjects<br>Search modes - Boolean/Phrase	19,027
S70	feedback	Expanders - Apply equivalent subjects<br>Search modes - Boolean/Phrase	44,435

S69	"self-monitoring"	Expanders - Apply equivalent subjects<br>Search modes - Boolean/Phrase	6,749
S68	remote	Expanders - Apply equivalent subjects<br>Search modes - Boolean/Phrase	19,070
S67	"individualised programme"	Expanders - Apply equivalent subjects<br>Search modes - Boolean/Phrase	13
S66	"individualized programme"	Expanders - Apply equivalent subjects<br>Search modes - Boolean/Phrase	9
S65	automated	Expanders - Apply equivalent subjects<br>Search modes - Boolean/Phrase	21,728
S64	tailored	Expanders - Apply equivalent subjects<br>Search modes - Boolean/Phrase	21,812
S63	"social network"	Expanders - Apply equivalent subjects<br>Search modes - Boolean/Phrase	9,739
S62	blog	Expanders - Apply equivalent subjects<br>Search modes - Boolean/Phrase	5,288
S61	tweet	Expanders - Apply equivalent subjects<br>Search modes - Boolean/Phrase	2,039
S60	twitter	Expanders - Apply equivalent subjects<br>Search modes - Boolean/Phrase	3,528
S59	IM	Expanders - Apply equivalent subjects<br>Search modes - Boolean/Phrase	14,035
S58	"instant message"	Expanders - Apply equivalent subjects<br>Search modes - Boolean/Phrase	15
S57	"chat room"	Expanders - Apply equivalent subjects<br>Search modes - Boolean/Phrase	112

S56	chat	Expanders - Apply equivalent subjects<br>Search modes - Boolean/Phrase	1,910
S55	bluetooth	Expanders - Apply equivalent subjects<br>Search modes - Boolean/Phrase	365
S54	SMS	Expanders - Apply equivalent subjects<br>Search modes - Boolean/Phrase	1,674
S53	"text messaging"	Expanders - Apply equivalent subjects<br>Search modes - Boolean/Phrase	3,938
S52	"text message"	Expanders - Apply equivalent subjects<br>Search modes - Boolean/Phrase	2,539
S51	ivr	Expanders - Apply equivalent subjects<br>Search modes - Boolean/Phrase	411
S50	"interactive voice response"	Expanders - Apply equivalent subjects<br>Search modes - Boolean/Phrase	1,260
S49	pda	Expanders - Apply equivalent subjects<br>Search modes - Boolean/Phrase	4,977
S48	"personal digital assistant"	Expanders - Apply equivalent subjects<br>Search modes - Boolean/Phrase	2,688
S47	"mobile device"	Expanders - Apply equivalent subjects<br>Search modes - Boolean/Phrase	739
S46	smartphone	Expanders - Apply equivalent subjects<br>Search modes - Boolean/Phrase	8,049
S45	"cellular phone"	Expanders - Apply equivalent subjects<br>Search modes - Boolean/Phrase	2,133
S44	"cell phone"	Expanders - Apply equivalent subjects<br>Search modes - Boolean/Phrase	2,173

S43	"mobile phone"	Expanders - Apply equivalent subjects<br>Search modes - Boolean/Phrase	3,470
S42	wireless	Expanders - Apply equivalent subjects<br>Search modes - Boolean/Phrase	14,362
S41	"computer-based"	Expanders - Apply equivalent subjects<br>Search modes - Boolean/Phrase	4,623
S40	computer	Expanders - Apply equivalent subjects<br>Search modes - Boolean/Phrase	146,014
S39	computers	Expanders - Apply equivalent subjects<br>Search modes - Boolean/Phrase	146,014
S38	"e-mail-based"	Expanders - Apply equivalent subjects<br>Search modes - Boolean/Phrase	52
S37	"electronic mail"	Expanders - Apply equivalent subjects<br>Search modes - Boolean/Phrase	2,414
S36	"website-based"	Expanders - Apply equivalent subjects<br>Search modes - Boolean/Phrase	42
S35	website	Expanders - Apply equivalent subjects<br>Search modes - Boolean/Phrase	23,030
S34	"web-based"	Expanders - Apply equivalent subjects<br>Search modes - Boolean/Phrase	16,001
S33	web	Expanders - Apply equivalent subjects<br>Search modes - Boolean/Phrase	133,908
S32	"internet-based"	Expanders - Apply equivalent subjects<br>Search modes - Boolean/Phrase	4,788
S31	"internet"	Expanders - Apply equivalent subjects<br>Search modes - Boolean/Phrase	69,734

S30	"phone-based"	Expanders - Apply equivalent subjects<br>Search modes - Boolean/Phrase	574
S29	"telephone-based"	Expanders - Apply equivalent subjects<br>Search modes - Boolean/Phrase	1,170
S28	"telephone"	Expanders - Apply equivalent subjects<br>Search modes - Boolean/Phrase	42,039
S27	"interactive media"	Expanders - Apply equivalent subjects<br>Search modes - Boolean/Phrase	194
S26	"mobile health"	Expanders - Apply equivalent subjects<br>Search modes - Boolean/Phrase	19,200
S25	"m-health"	Expanders - Apply equivalent subjects<br>Search modes - Boolean/Phrase	216
S24	"mhealth"	Expanders - Apply equivalent subjects<br>Search modes - Boolean/Phrase	16,972
S23	telehealth	Expanders - Apply equivalent subjects<br>Search modes - Boolean/Phrase	21,251
S22	(MH "Telemedicine+") OR (MH "Telerehabilitation") OR (MH "Telehealth") OR ""telemedicine""	Expanders - Apply equivalent subjects<br>Search modes - Boolean/Phrase	26,990
S21	""electronic health""	Expanders - Apply equivalent subjects<br>Search modes - Boolean/Phrase	34,378

S20	"e-health"	Expanders - Apply equivalent subjects<br>Search modes - Boolean/Phrase	1,489
S19	"Employment"	Expanders - Apply equivalent subjects<br>Search modes - Boolean/Phrase	68,291
S18	"Low education"	Expanders - Apply equivalent subjects<br>Search modes - Boolean/Phrase	1,948
S17	"Low income"	Expanders - Apply equivalent subjects<br>Search modes - Boolean/Phrase	20,727
S16	"Socio-economic inequity"	Expanders - Apply equivalent subjects<br>Search modes - Boolean/Phrase	3
S15	"Socioeconomic inequity"	Expanders - Apply equivalent subjects<br>Search modes - Boolean/Phrase	22
S14	"Socio-economic inequality"	Expanders - Apply equivalent subjects<br>Search modes - Boolean/Phrase	72
S13	"Socioeconomic inequality"	Expanders - Apply equivalent subjects<br>Search modes - Boolean/Phrase	311

S12	""Socioeconomic disparity""	Expanders - Apply equivalent subjects<br>Search modes - Boolean/Phrase	962
S11	""Socio-economic disparity""	Expanders - Apply equivalent subjects<br>Search modes - Boolean/Phrase	121
S10	""Economic inequity""	Expanders - Apply equivalent subjects<br>Search modes - Boolean/Phrase	69
S9	""Economic inequality""	Expanders - Apply equivalent subjects<br>Search modes - Boolean/Phrase	689
S8	(MH "Healthcare Disparities") OR (MH "Health Status Disparities") OR (MH "Economic Status") OR ""Economic disparity"" OR (MH "Economic Factors") OR (MH "Socioeconomic Factors")	Expanders - Apply equivalent subjects<br>Search modes - Boolean/Phrase	110,151
S7	""Social inequity""	Expanders - Apply equivalent subjects<br>Search modes - Boolean/Phrase	291
S6	"Social inequalit*"	Expanders - Apply equivalent subjects<br>Search modes - Boolean/Phrase	1,819
S5	""Social disparit*"" OR (MH "Social Class+") OR (MH "Social Support Index")	Expanders - Apply equivalent subjects<br>Search modes - Boolean/Phrase	13,362

S4	Adult	Expanders - Apply equivalent subjects<br>Search modes - Boolean/Phrase	1,422,495
S3	"Overweight"	Expanders - Apply equivalent subjects<br>Search modes - Boolean/Phrase	73,109
S2	"obese"	Expanders - Apply equivalent subjects<br>Search modes - Boolean/Phrase	41,687
S1	(MH "Obesity+") OR "Obesity" OR (MH "Obesity, Morbid")	Expanders - Apply equivalent subjects<br>Search modes - Boolean/Phrase	133,100
