# Supplementary material for: Effectiveness of eHealth weight management interventions in overweight and obese adults from low socioeconomic groups: a systematic review
Source: Syst Rev. 2023 Mar 30;12:59. doi: 10.1186/s13643-023-02207-3 (PMC10061957; doi:10.1186/s13643-023-02207-3)
Supplement: Supplementary file 3 — Additional file 3. Excluded Studies. A list of articles that may have appeared to meet the inclusion criteria, but which were excluded following full text review. [file 13643_2023_2207_MOESM3_ESM.pdf]

Excluded Articles Following Full Text Assessment

1. Alencar MK, Johnson K, Mullur R, Gray V, Gutierrez E, Korosteleva O. The efficacy of a telemedicine-based weight loss program with video conference health coaching support. Journal of Telemedicine and Telecare. 2017.

2. Almeida FA, You W, Harden SM, Blackman KCA, Davy BM, Glasgow RE, et al. Effectiveness of a worksite-based weight loss randomized controlled trial: the worksite study. Obesity (Silver Spring, Md). 2015;23(4):737-45.

3. Aschbrenner KA, Naslund JA, Shevenell M, Mueser KT, Bartels SJ. Feasibility of Behavioral Weight Loss Treatment Enhanced with Peer Support and Mobile Health Technology for Individuals with Serious Mental Illness. Psychiatric Quarterly. 2016;87(3):401-15.

4. Bader A, Gougeon R, Joseph L, Da Costa D, Dasgupta K. Nutritional education through Internet-delivered menu plans among adults with type 2 diabetes mellitus: Pilot study. Journal of Medical Internet Research. 2013;15(10):2525.

5. Baer HJ, De La Cruz BA, Nolido NV, John Orav E, Secinaro K, Rozenblum R, et al. Integrating an online weight management program with population health management support in primary care: Preliminary Results from the props study (partnerships for reducing overweight and obesity with patient-centered strategies). Journal of General Internal Medicine. 2019;34:S262-S3.

6. Baer HJ, De La Cruz BA, Rozenblum R, Nolido NV, Orav EJ, Secinaro K, et al. Effectiveness of an online weight management program integrated with population health management support in primary care: Results from the props study (partnerships for reducing overweight and obesity with patient-centered strategies). Journal of General Internal Medicine. 2020;35:S101.

7. Baillot A, Boissy P, Tousignant M, Langlois MF. Feasibility and effect of in-home physical exercise training delivered via telehealth before bariatric surgery. Journal of telemedicine and telecare. 2017;23(5):529-35.

8. Baltic R, Paskett ED, Lesko S, Kennedy S, Lengerich G, Roberto K, et al. Development and implementation of a church-based eHealth program to reduce obesity in Appalachian adults. Cancer Epidemiology Biomarkers and Prevention Conference: 7th AACR Conference on the Science of Health Disparities in Racial/Ethnic Minorities and the Medically Underserved San Antonio, TX United States Conference Publication:. 2015;24(10).

9. Bennett GG, Warner ET, Glasgow RE, Askew S, Goldman J, Ritzwoller DP, et al. Obesity treatment for socioeconomically disadvantaged patients in primary care practice. Archives of internal medicine. 2012;172(7):565-74.

10. Blom EE, Aadl, E, Solbraa AK, Oldervoll LM. Healthy Life Centres: A 3-month behaviour change programme's impact on participants' physical activity levels, aerobic fitness and obesity: An observational study. BMJ Open. 2020;10(35888).

11. Bosak KA, Yates B, Pozehl B. Effects of an internet physical activity intervention in adults with metabolic syndrome. Western Journal of Nursing Research. 2010;32(1):5-22.

12. Boutelle KN, Dubbert P, er Weg M. A pilot study evaluating a minimal contact telephone and mail weight management intervention for primary care patients. Eating and weight disorders : EWD. 2005;10(1):e1-5.

13. Bruno M, Touger-Decker R, Byham-Gray L, Denmark R. Workplace weight loss program: impact on quality of life. Journal of occupational and environmental medicine. 2011;53(12):1396-403.

14. Burke L, Jancey J, Howat P, Lee A, Kerr D, Shilton T, et al. Physical activity results of a home-based physical activity and nutrition program for seniors (PANS). Journal of Science and Medicine in Sport. 2012;15:S47-S8.

15. Cadmus-Bertram LA, Marcus BH, Patterson RE, Parker BA, Morey BL. Randomized Trial of a Fitbit-Based Physical Activity Intervention for Women. American Journal of Preventive Medicine. 2015;49(3):414-8.

16. Carpenter KM, Lovejoy JC, Lange JM, Hapgood JE, Zbikowski SM. Outcomes and utilization of a low intensity workplace weight loss program. Journal of obesity. 2014;2014:414987.

17. Chambliss HO, Huber RC, Finley CE, McDoniel SO, Kitzman-Ulrich H, Wilkinson WJ. Computerized self-monitoring and technology-assisted feedback for weight loss with and without an enhanced behavioral component. Patient education and counseling. 2011;85(3):375-82.

18. Chambliss HO, Huber RC, Finley CE, McDoniel SO, Kitzman-Ulrich H, Wilkinson WJ. Computerized self-monitoring and technology-assisted feedback for weight loss with and without an enhanced behavioral component. Patient Education & Counseling. 2011;85(3):375-82.

19. Chang M-W, Nitzke S, Brown R. Design and outcomes of a Mothers In Motion behavioral intervention pilot study. Journal of nutrition education and behavior. 2010;42(3):S11-21.

20. Cheung KL, Schwabe I, Walthouwer MJL, Oenema A, Lechner L, De Vries H. Effectiveness of a video-versus text-based computer-tailored intervention for obesity prevention after one year: A randomized controlled trial. International Journal of Environmental Research and Public Health. 2017;14(1275).

21. Chung LMY, Law QPS, Fong SSM, Chung JWY. Teledietetics improves weight reduction by modifying eating behavior: a randomized controlled trial. Telemedicine journal and e-health : the official journal of the American Telemedicine Association. 2014;20(1):55-62.

22. Cleghorn C, Wilson N, Nair N, Kvizhinadze G, Nghiem N, McLeod M, et al. Health benefits and cost savings of two weight loss interventions: Dietary counselling by practice nurses and promoting smartphone weight loss apps. Annals of Nutrition and Metabolism. 2017;71:795-6.

23. Colkesen EB, Niessen MA, Peek N, Vosbergen S, Kraaijenhagen RA, Van Kalken CK, et al. Initiation of health-behaviour change among employees participating in a web-based health risk assessment with tailored feedback. Journal of Occupational Medicine and Toxicology. 2011;6(5).

24. Cox K, Beilin L, Burke V. Predictors of physical activity and fitness in a 6-month, home-based physical activity program for older adults: MOVES. Journal of Science and Medicine in Sport. 2014;1:e68-e9.

25. Damschroder LJ, Lutes LD, Kirsh S, Kim HM, Gillon L, Holleman RG, et al. Small-changes obesity treatment among veterans: 12-Month outcomes. American Journal of Preventive Medicine. 2014;47(5):541-53.

26. Das SK, Brown C, Urban LE, O'Toole J, Gamache MMG, Weerasekara YK, et al. Weight loss in videoconference and in-person iDiet weight loss programs in worksites and community groups. Obesity (Silver Spring, Md). 2017;25(6):1033-41.

27. Dekkers JC, van Wier MF, Ariens GA, Hendriksen IJ, Pronk NP, Smid T, et al. Comparative effectiveness of lifestyle interventions on cardiovascular risk factors among a Dutch overweight working population: a randomized controlled trial. BMC public health. 2011;11(1):49.

28. Dossett ML, Chad-Friedman E, Pearsall M, Denninger JW, Wheeler AE, Miller KM, et al. Total lifestyle coaching: A pilot study evaluating a telephone coaching program on weight loss and behavioral eating habits in obese adults at a community health center. Journal of Alternative and Complementary Medicine. 2016;22:A73.

29. Dunn C, Whetstone LM, Kolasa KM, Jayaratne KSU, Thomas C, Aggarwal S, et al. Using Synchronous Distance-Education Technology to Deliver a Weight Management Intervention. Journal of Nutrition Education and Behavior. 2014;46(6):602-9.

30. Earnest CP, Church TS. Evaluation of a Voluntary Worksite Weight Loss Program on Metabolic Syndrome. Metabolic syndrome and related disorders. 2015;13(9):406-14.

31. Estabrooks PA, Wilson KE, McGuire TJ, Harden SM, Ramalingam NP, Schoepke L, et al. A Quasi-Experiment to Assess the Impact of a Scalable, Community-Based Weight Loss Program: Combining Reach, Effectiveness, and Cost. Journal of General Internal Medicine. 2017;32:24-31.

32. Faghri PD, Omokaro C, Parker C, Nichols E, Gustavesen S, Blozie E. E-technology and pedometer walking program to increase physical activity at work. The journal of primary prevention. 2008;29(1):73-91.

33. Ganesan AN, Louise J, Horsfall M, Bilsborough SA, Hendriks J, McGavigan AD, et al. International Mobile-Health Intervention on Physical Activity, Sitting, and Weight: The Stepathlon Cardiovascular Health Study. Journal of the American College of Cardiology. 2016;67(21):2453-63.

34. Godino JG, Golaszewski NM, Norman GJ, Rock CL, Griswold WG, Arredondo E, et al. Text messaging and brief phone calls for weight loss in overweight and obese English-and Spanish-speaking adults: A 1-year, parallel-group, randomized controlled trial. PLoS Medicine. 2019;16.

35. Gussenhoven AHM, van Wier MF, Bosmans JE, Dekkers JC, van Mechelen W. Cost-effectiveness of a distance lifestyle counselling programme among overweight employees from a company perspective, ALIFE@Work: a randomized controlled trial. Work (Reading, Mass). 2013;46(3):337-46.

36. Haas K, Hayoz S, Maurer-Wiesner S. Effectiveness and feasibility of a remote lifestyle intervention by dietitians for overweight and obese adults: Pilot study. Journal of Medical Internet Research. 2019;21(4):e11664.

37. Han MK, Cho B, Kwon H, Son KY, Lee H, Lee JK, et al. A Mobile-Based Comprehensive Weight Reduction Program for the Workplace (Health-On): Development and Pilot Study. JMIR mHealth and uHealth. 2019;7(11):e11158.

38. Harden SM, You W, Almeida FA, Hill JL, Linnan LA, Allen KC, et al. Does Successful Weight Loss in an Internet-Based Worksite Weight Loss Program Improve Employee Presenteeism and Absenteeism? Health education & behavior : the official publication of the Society for Public Health Education. 2015;42(6):769-74.

39. Jaime PC, oni DH, Sarno F. Impact of an education intervention using email for the prevention of weight gain among adult workers. Public health nutrition. 2014;17(7):1620-7.

40. Jakicic JM, Davis KK, Rogers RJ, King WC, Marcus MD, Helsel D, et al. Effect of wearable technology combined with a lifestyle intervention on long-term weight loss: The IDEA randomized clinical trial. JAMA - Journal of the American Medical Association. 2016;316(11):1161-71.

41. Joseph RP, Cherrington A, Allison JJ, Kim YI, Durant NH. Results of the love your heart study: A culturallyadapted internet-enhanced physical activity promotion program for overweight and obese African American college females. Journal of Adolescent Health. 2014;1:S68.

42. Kassim MSA, Manaf MRA, Nor NSM, Ambak R. Effects of lifestyle intervention towards obesity and blood pressure among housewives in klang valley: A quasi-experimental study. Malaysian Journal of Medical Sciences. 2017;24(6):83-91.

43. Kerr J, Patrick K, Norman G, Stein MB, Calfas K, Zabinski M, et al. Randomized control trial of a behavioral intervention for overweight women: impact on depressive symptoms. Depression & Anxiety (1091-4269). 2008;25(7):555-8.

44. Kirkman MS, Weinberger M, sman PB, Samsa GP, Shortliffe EA, Simel DL, et al. A telephone-delivered intervention for patients with NIDDM: Effect on coronary risk factors. Diabetes Care. 1994;17(8):840-6.

45. Kozica SL, Lombard CB, Ilic D, Ng S, Harrison CL, Teede HJ. Acceptability of delivery modes for lifestyle advice in a large scale randomised controlled obesity prevention trial. BMC public health. 2015;15:699.

46. Kurtzman GW, Day SC, Small DS, Lynch M, Zhu J, Wang W, et al. Social Incentives and Gamification to Promote Weight Loss: The LOSE IT Randomized, Controlled Trial. Journal of general internal medicine. 2018;33(10):1669-75.

47. Lin M, Mahmooth Z, Dedhia N, Frutchey R, Mercado CE, Epstein DH, et al. Tailored, Interactive Text Messages for Enhancing Weight Loss among African American Adults: The TRIMM Randomized Controlled Trial. American Journal of Medicine. 2015;128(8):896-904.

48. Lison JF, Palomar G, Mensorio MS, R.M BA, Cebolla-Marti A, Botella C, et al. Impact of a Web-Based Exercise and Nutritional Education Intervention in Patients Who Are Obese with Hypertension: Randomized Wait-List Controlled Trial. Journal of Medical Internet Research. 2020;22.

49. Louis Walthouwer MJ, Oenema A, Lechner L, De Vries H. Comparing a video and text version of a web-based computer-tailored intervention for obesity prevention: A randomized controlled trial. Journal of Medical Internet Research. 2015;17(10):e236.

50. Lutes L, Damschroder L, Masheb R, Kim H, Gillon L, Holleman R, et al. Behavioral Treatment for Veterans with Obesity: 24-Month Weight Outcomes from the ASPIRE-VA Small Changes Randomized Trial. JGIM: Journal of General Internal Medicine. 2017;32:40-7.

51. Marcus BH, Hartman SJ, Larsen BA, Pekmezi D, Dunsiger SI, Linke S, et al. Pasos Hacia La Salud: a randomized controlled trial of an internet-delivered physical activity intervention for Latinas. The international journal of behavioral nutrition and physical activity. 2016;13:62.

52. Patrick K, Calfas KJ, Norman GJ, Rosenberg D, Zabinski MF, Sallis JF, et al. Outcomes of a 12-month web-based intervention for overweight and obese men. Annals of Behavioral Medicine. 2011;42(3):391-401.

53. Pekmezi D, Ainsworth C, Joseph RP, Williams V, Desmond R, Meneses K, et al. Pilot Trial of a Home-based Physical Activity Program for African American Women. Medicine and Science in Sports and Exercise. 2017.

54. Pekmezi DW, Neighbors CJ, Lee CS, Gans KM, Bock BC, Morrow KM, et al. A culturally adapted physical activity intervention for Latinas: a randomized controlled trial. American Journal of Preventive Medicine. 2009;37(6):495-500.

55. Pellegrini CA, Verba SD, Otto AD, Helsel DL, Davis KK, Jakicic JM. The comparison of a technology-based system and an in-person behavioral weight loss intervention. Obesity. 2012;20(2):356-63.

56. Pressler A, Knebel U, Esch S, Kolbl D, Esefeld K, Scherr J, et al. An internet-delivered exercise intervention for workplace health promotion in overweight sedentary employees: a randomized trial. Preventive medicine. 2010;51(3):234-9.

57. Robroek SJ, Polinder S, Bredt FJ, Burdorf A. Cost-effectiveness of a long-term Internet-delivered worksite health promotion programme on physical activity and nutrition: a cluster randomized controlled trial. Health education research. 2012;27(3):399-410.

58. Ryu H, Jung J, Cho J, Chin DL. Program development and effectiveness of workplace health promotion program for preventing metabolic syndrome among office workers. International Journal of Environmental Research and Public Health. 2017;14(878).

59. Schroder KEE. Computer-assisted dieting: effects of a randomised controlled intervention. Psychology & health. 2010;25(5):519-34.

60. Siriwoen R, Chongsuwat R, Tansakul S, Siri S. Effectiveness of a Weight Management Program Applying Mobile Health Technology as a Supporting Tool for Overweight and Obese Working Women. Asia-Pacific Journal of Public Health. 2018;30(6):572-81.

61. Sorensen G, Stoddard A, Quintiliani L, Ebbeling C, Nagler E, Yang M, et al. Tobacco use cessation and weight management among motor freight workers: results of the gear up for health study. Cancer causes & control : CCC. 2010;21(12):2113-22.

62. Steinberg DM, Levine EL, Lane I, Askew S, Foley PB, Puleo E, et al. Adherence to self-monitoring via interactive voice response technology in an eHealth intervention targeting weight gain prevention among Black women: randomized controlled trial. Journal of medical Internet research. 2014;16(4):e114.

63. Sun A, Cheng J, Bui Q, Liang Y, Ng T, Chen J-L. Home-Based and Technology-Centered Childhood Obesity Prevention for Chinese Mothers With Preschool-Aged Children. Journal of transcultural nursing : official journal of the Transcultural Nursing Society. 2017;28(6):616-24.

64. Takada A, Nakamura R, Furukawa M, Takahashi Y, Nishimura S, Kosugi S. The relationship between weight loss and time and risk preference parameters: a randomized controlled trial. Journal of biosocial science. 2011;43(4):481-503.

65. Tate DF, Wing RR, Winett RA. Using internet technology to deliver a behavioral weight loss program. Journal of the American Medical Association. 2001;285(9):1172-7.

66. Terry PE, Seaverson ELD, Grossmeier J, Anderson DR. Effectiveness of a worksite telephone-based weight management program. American journal of health promotion : AJHP. 2011;25(3):186-9.

67. Turnin MC, Bourgeois O, Cathelineau G, Leguerrier AM, Halimi S, re-Banon D, et al. Multicenter randomized evaluation of a nutritional education software in obese patients. Diabetes & metabolism. 2001;27(2):139-47.

68. Vadheim LM, McPherson C, Kassner DR, erwood KK, Hall TO, Butcher MK, et al. Adapted diabetes prevention program lifestyle intervention can be effectively delivered through telehealth. The Diabetes educator. 2010;36(4):651-6.

69. Van Genugten L, Van Empelen P, Oenema A. Intervention use and action planning in a web-based computer-tailored weight management program for overweight adults: Randomized controlled trial. Journal of Medical Internet Research. 2014;16(7).

70. VanWormer JJ, Martinez AM, Martinson BC, Crain AL, Benson GA, Cosentino DL, et al. Self-Weighing Promotes Weight Loss for Obese Adults. American Journal of Preventive Medicine. 2009;36(1):70-3.

71. Ventura Marra M, Lilly CL, Nelson KR, Woofter DR, Malone J. A Pilot Randomized Controlled Trial of a Telenutrition Weight Loss Intervention in Middle-Aged and Older Men with Multiple Risk Factors for Cardiovascular Disease. Nutrients. 2019;11(2):229.

72. Viglione C, Bouwman D, Rahman N, Fang Y, Beasley JM, Sherman S, et al. A technology-assisted health coaching intervention vs. enhanced usual care for Primary Care-Based Obesity Treatment: A randomized controlled trial. BMC Obesity. 2019;6(4).

73. West DS, Harvey JR, Krukowski RA, Prewitt TE, Priest J, Ashikaga T. Do individual, online motivational interviewing chat sessions enhance weight loss in a group-based, online weight control program? Obesity (Silver Spring, Md). 2016;24(11):2334-40.

74. Wilson MG, DeJoy DM, enberg RJ, Corso P, Padilla H, Zuercher H. Effect of Intensity and Program Delivery on the Translation of Diabetes Prevention Program to Worksites: A Randomized Controlled Trial of Fuel Your Life. Journal of occupational and environmental medicine. 2016;58(11):1113-20.

75. Winett RA, Anderson ES, Wojcik JR, Winett SG, Moore S, Blake C. Guide to health: A randomized controlled trial of the effects of a completely web-based intervention on physical activity, fruit and vegetable consumption, and body weight. Translational Behavioral Medicine. 2011;1(1):165-74.

76. Wing RR, Becofsky K, Wing EJ, McCaffery J, Boudreau M, Evans EW, et al. Behavioral and Cardiovascular Effects of a Behavioral Weight Loss Program for People Living with HIV. AIDS and Behavior. 2019.

77. Yancy WS, Shaw PA, Reale C, Hilbert V, Yan J, Zhu J, et al. Effect of Escalating Financial Incentive Rewards on Maintenance of Weight Loss: A Randomized Clinical Trial. JAMA Network Open. 2019;2(11):e1914393-e.
